# An Australian childhood obesity summit: the role of data and evidence in 'public' policy making

**DOI:** 10.1186/1743-8462-2-17

**Published:** 2005-07-20

**Authors:** Nathan SA, Develin E, Grove N, Zwi AB

**Affiliations:** 1 School of Public Health and Community Medicine, The University of New South Wales, Sydney NSW 2052 Australia; 2 Centre for Chronic Disease Prevention and Health Advancement, NSW Department of Health, Locked Mail Bag 961, North Sydney NSW 2059, Australia

## Abstract

**Background:**

Overweight and obesity in Australia has risen at an alarming rate over the last 20 years as in other industrialised countries around the world, yet the policy response, locally and globally, has been limited. Using a childhood obesity summit held in Australia in 2002 as a case study, this paper examines how evidence was used in setting the agenda, influencing the Summit debate and shaping the policy responses which emerged. The study used multiple methods of data collection including documentary analysis, key informant interviews, a focus group discussion and media analysis. The resulting data were content analysed to examine the types of evidence used in the Summit and how the state of the evidence base contributed to policy-making.

**Results:**

Empirical research evidence concerning the magnitude of the problem was widely reported and largely uncontested in the media and in the Summit debates. In contrast, the evidence base for action was mostly opinion and ideas as empirical data was lacking. Opinions and ideas were generally found to be an acceptable basis for agreeing policy action coupled with thorough evaluation. However, the analysis revealed that the evidence was fiercely contested around food advertising to children and action agreed was therefore limited.

**Conclusion:**

The Summit demonstrated that policy action will move forward in the absence of strong research evidence. Where powerful and competing groups contest possible policy options, however, the evidence base required for action needs to be substantial. As with tobacco control, obesity control efforts are likely to face ongoing challenges around the nature of the evidence and interventions proposed to tackle the problem. Overcoming the challenges in controlling obesity will be more likely if researchers and public health advocates enhance their understanding of the policy process, including the role different types of evidence can play in influencing public debate and policy decisions, the interests and tactics of the different stakeholders involved and the part that can be played by time-limited yet high profile events such as Summits.

## Background

Any policy-making process is complex – it deals with human and political dynamics, the use of resources, and power [1]. The development and implementation of policy in a democracy seeks to meet multiple objectives [2]: addressing major health and social policy problems, using public resources wisely, satisfying a range of stakeholders, avoiding conflict, and ensuring that political and economic objectives are met. Research is only one influence in the ongoing process of policy-making [3]. In setting the agenda, formulating policy, and implementing and evaluating it, various forms of evidence are sought and utilised. While conventionally such evidence is conceived as being derived from "scientific and objective" research, it is increasingly clear that a much wider range of sources and forms of evidence are influential [4]. There has been significant debate in Australia about the interface between evidence and policy-making [5], but little detailed analysis of the way evidence shapes the process of policy-making. This paper examines the role of data and evidence in public policy-making in response to childhood obesity in an Australian state, New South Wales (NSW).

Overweight and obesity (O&O) in Australia, as in many countries, has risen at an alarming rate over the last 20 years. Overweight is classified as a body mass index (BMI) of 25 and above, and obesity as 30 and above [6]. Obesity in men in Australia rose from 9.3% in 1980 to 17.1% in 2000 and for women from 8.0% to 18.9% [7,8]. O&O in children and young people has also increased markedly. From 1985 to 1995 the level of combined O&O in children more than doubled in all but the youngest age group of boys whilst the level of obesity tripled in all age groups and for both sexes [9].

Despite rising obesity, the policy response has been limited and hampered by a lack of evidence concerning effective interventions. The World Health Organisation (WHO) has highlighted "globesity" and released the *Global Strategy on Diet, Physical Activity and Health *[10]. Earlier, the United States (US) Surgeon General's *Call to Action *emphasised the need to create supportive environments which provide accessible and affordable healthy food choices and convenient opportunities for regular physical activity [11].

Australia was one of the first countries to produce an integrated national strategy for the prevention of O&O. The National Health and Medical Research Council (NHMRC) report *'Acting on Australia's weight: a strategic plan for the prevention of overweight and obesity' *[12], was released in 1997, but its recommendations, which included strategies such as promoting physical activity, dietary monitoring, and encouraging the development of school canteen policies remained largely unaddressed.

Within this environment characterised by public policy inertia, the issue of childhood obesity, and the need for effective interventions, was brought to the forefront of one Australian state government's agenda through the NSW Childhood Obesity Summit (hereafter referred to as 'the Summit') in 2002. While obesity had already been identified as a problem, how to respond was unclear. With little evidence available to guide Government responses to the issue, the state health department's (hereafter referred to as NSW Health) articulated purpose of the Summit was to i) create better understanding in the community; ii) inform Members of Parliament; iii) hear and consider the views of families, parents and young people; iv) examine existing approaches and consider new ideas in a bipartisan forum; v) consider evidence; vi) identify ways to improve existing strategies and services; vii) build community consensus about future directions, and viii) recommend a future course of action so that the best available strategies, both long and short term, would be implemented to overcome the childhood overweight and obesity problem [13]. This paper examines the role of evidence and data in entrenching childhood obesity on the policy agenda, in shaping the Summit debate and informing the outcomes and the policies that were subsequently adopted.

## Methods

### Data collection

Data were collected from the transcripts of the Summit proceedings [14-16], media articles, the Summit *Communiqué *[17] which outlined the agreed resolutions, the *Government Action Plan *[18] published after the Summit and the announcement by the NSW Health Minister in December 2002 [19]. 'Factiva', a searchable archive of print media, was used to identify articles that referred to childhood obesity in the four main NSW statewide newspapers and one national newspaper (the Daily Telegraph, Sunday Telegraph, Sydney Morning Herald, The Sun Herald and the Australian) in the three months prior to the announcement of the Summit in July 2002 until the first public response from government in December 2002. There were 127 articles retrieved from this search.

Seven semi-structured key informant interviews [20] and one focus group discussion (FGD)[20] with three health staff involved in the Summit's organization were also conducted. The key informants included NSW health staff and experts in human nutrition, physical activity, and population health. The interviews and focus group discussion used a guide to elicit opinions on the stimulus for, and organization of, the Summit and its outcomes. The focus group discussion was transcribed for analysis and the interviews were used as background material.

### Data analysis

The transcripts of the Summit proceedings [14-16], media articles and other key documents were reviewed and content analysed [20] to examine what type of evidence was used, by whom (eg. experts, industry, advocates) and for what purpose. Evidence that was valued or contested in the Summit debates and the media coverage received particular attention. The type of evidence used was categorised into three types based on a model adapted from Bowen & Zwi [4] who outlined five types of evidence. The categorisation used in the current study were empirical research (Type 1), such as randomised controlled trials, case control and cohort studies, time series analyses, observational studies, case reports and qualitative studies; ideas and opinions (Type 2) which incorporated the two categories of 'knowledge and information' and 'ideas and interests' outlined by Bowen & Zwi, and included evidence such as the results of consultation processes, opinions and views of "experts", interest groups and community members; and economic data (Type 3) which focused on economic evaluation, finance and resource implications.

### Rigour

Rigour was addressed through triangulation, clear exposition of methods and reflexivity [21].

Triangulation is the use of different approaches, such as interviews and document analysis, to answer the same question which strengthens the rigour of a study and the interpretations made [22]. In the current study, interviews, analysis of transcripts from the summit debate and related documents and media coverage were used to answer questions posed in relation to the role of evidence in the NSW Childhood Obesity Summit.

It is important to consider the ways in which researchers and authors' past and present experiences may have shaped the way data was collected and interpreted – often referred to as reflexivity [22]. All the authors of this paper are involved, at some level, in public health advocacy and support a range of initiatives to address public health problems, including childhood obesity. The paper arose from a desire by the authors to better understand and reflect upon the role of evidence and its use by the different stakeholders in the Summit debate and how the debates around evidence were seen to influence the resolutions agreed. The involvement of all authors in the analysis and interpretation of the data presented in this paper, data triangulation, clear exposition of methods, conduct of a focus group with some of the key actors involved, and reflection on alternate ways of viewing the data were all important in enhancing the rigour of the study and the credibility of the interpretations made [22,23].

## Results

Three phases were discernible in the process of policy making that occurred as part of the NSW Childhood Obesity Summit: 1) building and maintaining the momentum 2) summit debate and 3) outcomes and policy formulation.

### 1) Building and maintaining the momentum

Obesity had been recognised as a longstanding and increasingly important public health problem. Ebbeling et al (2002) pointed to publications decades earlier highlighting the issue and the need for a policy response[24]. Media interest in the issue of obesity in Australia was stimulated by available data highlighting *"the doubling and tripling" *of rates of obesity and concerns around the *"second fattest kids in the world" (FGD)*. Obesity was seen as *"the new tobacco" *– the public health issue which was being recognised as demanding attention. Articles published in the peer review literature around this time [24,25] were triggers for media coverage and interviews with key informants and the focus group with NSW Health staff all emphasised the importance of media coverage in bringing the issue to public and policy attention:

"It [media coverage] was partly driven by data...the MJA [Medical Journal of Australia] also carried some data on childhood obesity and ... reinterpreting existing data sets. ...so that put it on the radar, that doesn't mean you've got [a] Summit happening yet... the data is essential – it is necessary, but not sufficient." (FGD)

"The doubling and tripling was the most used [news] grab everywhere, in every article, and it is still used." (FGD)

Why was NSW Health interested? The issue was shown to be important to the public. It provided the opportunity to divert attention away from other health issues which are considered solely the responsibility of government, for example, health care service provision. NSW Health also wanted to show leadership in an area where there was arguably Federal Government inaction. In New South Wales there was a clear perception that *"prior to the Summit there was a national leadership vacuum" *around childhood obesity *(FGD)*. An earlier government summit on illicit drugs [26], had mobilised massive public attention and resources and it was hoped by NSW Health that a childhood obesity summit would draw in funds and resources to address this public health problem. A summit was seen as providing scope to debate interventions in an area where there was no scientific or political clarity at the time:

"there was interest, we were asked to do things, write things, pull things together... there were lots of false starts...we had things in train that were going to take another 5 or 10 years and they said they wanted a solution today... a summit was suggested as a way forward."(FGD)

Table 1 shows the number of media articles by month between April 2002 and December 2002. Within each month the percentage of articles that used Type 1, Type 2 or Type 3 evidence are identified. All the articles drew on more than one type of evidence. Peak months of coverage were July when the Summit was announced (n = 15), September when the Summit was held (n = 40), and December when the Health Minister announced the preliminary government response (n = 19). In the months prior to the announcement of the Summit, childhood obesity was covered 1–2 times per week in the newspapers studied.

**Table 1:** Number of media articles and evidence type used

		**Number of articles**		
**Month**	**Total Articles**	**Type 1**	**Type 2**	**Type 3**

April	5	5	5	0
May	7	6	3	0
June	9	9	5	2
July^1^	15	7	4	0
August	10	6	3	0
September^2^	40	15	16	2
October	11	4	5	0
November	11	8	8	2
December^3^	19	12	12	3

^1^Announcement of Summit

^2 ^Month in which Summit was held

^3 ^Month in which government initial policy response was announced

In the lead up to the Summit, most of the articles cited evidence of at least one type concentrating on Type 1 evidence focussed on the magnitude of the problem, backed up by expert opinion (Type 2). In the month before the first announcement by government in December economic data (Type 3), always referring to the cost of obesity to the health care system, were also reported.

Prior to the Summit and throughout the study period, Type 1 evidence was widely reported and largely uncontested, quoting authoritative sources such as the Lancet [24] and the Medical Journal of Australia [25] concerning the magnitude of the problem. Media representations drew on such data to present 'sound bites' to stimulate debate. The most commonly reported statistics were that either one in four, or one in 5 children in Australia was overweight or obese and that overweight and obesity had doubled between 1985 and 1995. These data from Magarey et al (2001) [9] were also contained in the background document prepared for the Summit [27] and included in the factual preamble to the Summit resolutions [16]. Once the Summit was underway, Type 2 data were more widely reported and ideas from experts, community members and key stakeholders concerning the way forward, were presented in the media. In putting forward their views, these stakeholders called on common sense understandings, research studies or pointed to a lack of conclusive evidence to support inaction.

Food advertising to children was a case in point. Prior to the Summit, debates about evidence in the media focused on taxing 'high fat foods' and banning food advertising to children. The soft drink industry spoke about the lack of good evidence for the effectiveness of such initiatives and the negative economic impact of a "fat tax". Physical activity and the role of parents as an influence on obesity were highlighted by the advertising and food industries as being the major influences on childhood obesity. Results from Sweden which were stated by the food and advertising industry as showing obesity rising despite an advertising ban were used to demonstrate that *"there is no evidence that advertising makes children eat more fatty foods" (The Australian Newspaper, 1 July 2002*)[28]. It became clear from the media coverage during the Summit that a ban on food advertising was the critical concern for industry who were calling a 'clear link' between harmful childhood behaviour and commercials, with editorials suggesting that instead *"parents are the dominant influence on food choices" (Daily Telegraph, 12 September 2002) *[29].

### 2) Summit debate

The Summit was held in September 2002 at Parliament House in NSW. An across-government organising committee oversaw delegate selection and sought to ensure balanced representation including: i) children and young people; ii) families, parents and community perspectives; iii) experts; iv) relevant peak bodies; v) special population perspectives, such as the socially disadvantaged, people from culturally and linguistically diverse communities, Aboriginal and Torres Strait Islanders, rural and remote communities, and people with disabilities.

The Summit provided an opportunity for delegates to present their case for action during plenary sessions. During the Summit, nine working groups (WGs) were convened: i) Early Childhood, ii) Family and Community, iii) School Education, iv) Health, v) Sport, Recreation & Fitness, vi) Local Government, vii) Commercial Food Industry, viii) Media, and ix) Transport and Planning.

The WGs were requested to put forward 10–15 resolutions for the Communiqué to be presented to government. The importance of evidence for the resolutions was made clear by the NSW Premier on the first day of the summit when he referred to the NSW Drug Summit [26], which had been held in May 1999.

"The Drug Summit emphasised looking at evidence, basing policies on evidence ... I would like that to be your guide too." (NSW Premier, Day 1 pg 33)

The case for action to tackle childhood obesity was uncontested from the outset. In opening the Summit the NSW Minister for Health referred to strong empirical evidence of the magnitude of childhood obesity:

"In Australia the level of combined overweight and obesity between 1985 and 1995 has more than doubled... Today in New South Wales one in five children aged between seven and 15 are classified as being either overweight or obese." (NSW Minister for Health, Day 1, pg 5)

Experts from government departments, academic institutions and the health service put forward similar statistics that highlighted the magnitude of obesity. Many outlined the consequences of overweight and obesity for type 2 diabetes in particular. The use of simple statistical concepts such as *"doubling in rates" *and *"one in five of our children" *were commonplace. Economic evidence highlighted the cost of the *"obesity epidemic" *to society – *"it costs us a community $830 million a year" (Minister for Health, Day 1, pg 7) *and individuals – *"in one year the personal cost to individuals who are obese is $19 billion" (expert, Day 1 pg 16)*. Such data were uncritically and widely accepted during the Summit.

On the opening day, experts, parents, community groups and industry talked anecdotally about societal changes over decades and their impact on physical activity and food consumption. Statistics and studies were referred to in support of these observations, such as an increased reliance on carbonated sugared drinks, although no actual data were provided. Data from the US concerning changes in levels of physical activity were presented: *"most people in my generation walked to school, today less than a third of children in the United States walk to school" (US expert, Day 1, pg 12) *and Australian data on sedentary activity: *"97% of our adolescents watch television ... between 60 and 80% play computer or video games." (Australian expert, Day 1, pg 16)*. Anecdotal observations about changing societal behaviours and environments were widely cited and seen as important factors to address despite the lack of reliable trend data and research evidence:

"we do not yet have evidence that any single one of these factors is driving the epidemic" (US expert, Day 1, pg 13)

"we know very little in any, firm solid way about the factors that influence young people to be active or sedentary – all we have to work with over the next three days are some recently informed guesses and some far less well-informed speculations" (Australian expert, Day 1, pg 12)

The views of young people, were an integral part of the Summit process and provided an emotive appeal to take action. Young people's stories were shown on video and they addressed the Summit. However, there appeared to be little attempt to draw these views together, articulate common threads or examine whether and how such views related to other empirical data and expert opinion. A young person opening the Summit stated that *"It is genuinely important that our voice be heard"(young person, Day 1, pg 2)*. The FGD participants saw young people's stories as being powerful in stimulating action:

" we had to have lots of consultation processes that included the voice of the child... engaging the children... it was the most powerful thing." (FGD)

A young person at the Summit, however, expressed frustration about the focus on evidence:

"Unfortunately we have been bombarded with statistics. They have been repeated over and over again ... we are almost scared to put up a decent suggestion." (young person, Day 2, pg 30)

In contrast to the research evidence supporting the magnitude of the problem and the influencing factors, evidence supporting calls for action were mostly opinion and ideas with some reference to overseas efforts. Nonetheless, much was made of the need for evidence-based strategies, with a US expert claiming three strategies that were *"defensible, but not conclusive" (US expert, Day 1, pg 13)*: breastfeeding, limiting television viewing and the promotion of physical activity. A Cochrane systematic review [30] covering 1985–2001 and encompassing 14,000 studies was reported (researcher, Day 1, pg 37). It found 11 studies of a high enough quality to examine the effectiveness of the intervention and it found only small or no effects with those interventions that were most effective focussed on reducing sedentary behaviour. A few delegates questioned the need for evidence from primary prevention trials, pointing to broader experiences that tell us "what works". They highlighted other successful public health campaigns such as in tobacco control as evidence for the success of a range of strategies, including advertising controls and taxes:

"we do need evidence, we do need to work at what has been shown to be the most effective, but that should not inhibit us from acting now. There have been a number of successful public health programs that have been introduced without definitive evidence." (expert, Day 1, pg 39)

The FGD and comments by Summit delegates highlighted the need for action coupled with thorough evaluation:

"There needs to be a recognition of the sense of urgency...that policy won't wait for the data." (FGD)

"This is about promising interventions, we have to just go with promising interventions, make sure they do no harm and just evaluate the heck out of them, and then maybe in ten years time, if they weren't the best things to do, well at least we did something" (FGD)

"we need periodic surveys to tell us how we are doing with respect to implementation of strategies ... we need causal models, that is, longitudinal studies which allow us to link risk factors like change in the food supply with changes in the prevalence of obesity." (US expert, Day 1, pg 13)

"we would like to see a regular – maybe five yearly – national nutrition, physical activity and health survey." (industry, Day 2, pg 2)

"Certainly, we need to take action, but at the same time we need to be doing research. We cannot continue to act in an evidence vacuum." (expert, Day 2, pg 20)

**Figure 1:**
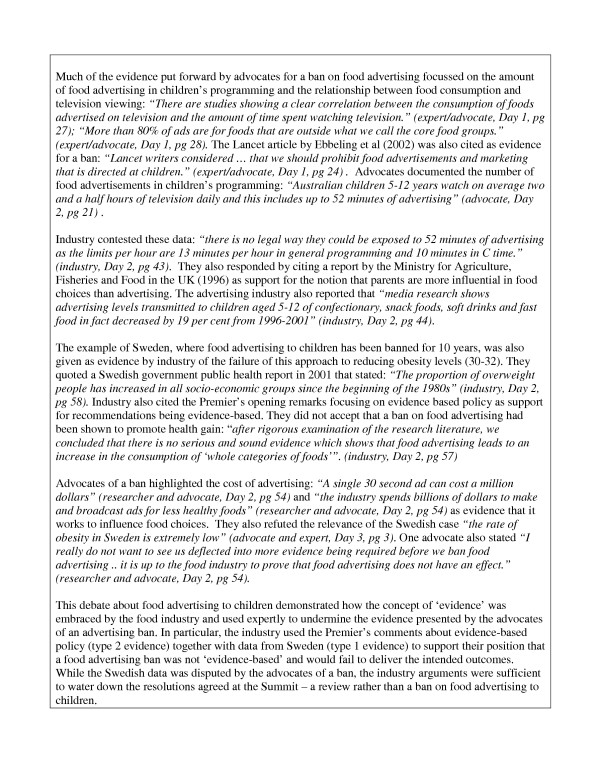
Contesting the evidence: food advertising and obesity.

The most contentious issue centred on the role of food advertising to children (see Figure 1). The intensity of the debate between food industry representatives and the advocates of a ban on food advertising to children clearly illustrates the way different types of evidence are drawn upon to articulate a particular position or undermine that of opposing perspectives.

### 3) Outcomes and policy formulation

The final Communiqué to government was to include a "factual foundation" and recommendations and resolutions for future action. The purpose of this component of the Summit was to: *"Frame evidence-based solutions within a community-based 'reality check' perspective."(Day 3, pg1)*

Evidence of the magnitude of overweight and obesity was included with little debate. Statements about the influencing factors were carefully worded to reflect agreement on importance and available evidence:

"Although physical activity trend data is lacking, it is apparent that children and adolescents are less physically active" (Day 2, pg 72)

"An increase in television viewing is associated with an increase in obesity in children. An increase in sedentary behaviour is associated with an increase in obesity in children. Experts have advised that television viewing needs to be one of the targets for obesity control efforts" (Day 2, pg 78)

Exposure to advertising messages was included in the factual preamble referring to the range of potential influences on food selection behaviours. The resolutions about food advertising to children generated the most debate concerning the evidence-base for such interventions (see Figure 1) and the relationship between food choice and television viewing. This debate illustrates the use of different types of evidence by industry representatives as one means of opposing calls for a ban on food advertising to children.

A resolution to ban food advertising to children was not agreed. In its place agreement was reached to have an independent review by the Federal government of the regulatory arrangements for food advertising *"in recognition that food advertising is one of the contributing factors to the prevalence of eating habits that may promote obesity"(Day 3, pg 9) *in addition to a review of a voluntary code to be undertaken by industry. Attempts by the Food Industry to have this statement deleted from the Communiqué were not successful. A systematic review of the impact of food advertising on diet, physical activity and childhood obesity was also recommended.

All other resolutions passed with minimal debate, including those addressing physical activity, school education, transport and planning. Most of the resolutions agreed at the Summit and taken up in the subsequent *Government Action Plan *[18] were focused on physical activity and nutrition education. Mandatory guidelines for school canteens also passed as a resolution despite some opposition from industry. Numerous resolutions in the Communiqué [17] referred to research and a detailed section on surveillance and monitoring proposed a funded collaborative centre of excellence in research, prevention and management.

Limited attention was devoted to the financial and logistical feasibility of the resolutions – this was apparent by the number of resolutions that required intervention at a federal rather than state level. However, the preliminary response from the government in December considered what was feasible in the current financial and political context:

"it wasn't really evidence-based, it was the feasibility of whatever strategy they had suggested..." (FGD)

In the final Summit address by the NSW Health Minister [16] the two resolutions specifically mentioned and strongly supported were the recommendations on school canteens and a collaborative centre for excellence for overweight and obesity research. These two initiatives were subsequently publicly announced in December as the key response to the Summit by the government [19]. The advisers to the Minister and NSW Health were concerned to ensure that the Summit resulted in some *"announceable" *interventions – and the two chosen seemed *"doable"*, of value, and in some respects least contentious *(FGD)*.

## Discussion

This paper has sought to present key elements of the use of data and evidence in the NSW Childhood Obesity Summit. There are other dimensions of policy-making which deserve attention and other interpretations of the process possible. As indicated by Ham and Hill "It is rarely possible to agree on one version of events: the most that can be achieved is a plausible interpretation" [2] (p xi).

Empirical evidence of the magnitude of the obesity problem and the economic cost to the health system were critical to generating publicity and framing the case for action on childhood obesity in the lead up and during the Summit. This evidence was never contested and became a part of the factual foundation of the Summit Communiqué [17]. It is clear that the combination of Type I data, which was largely epidemiological in origin, and Type 3 data about the economic costs of the problem was persuasive.

The lack of empirical evidence for many of the influencing factors and related interventions, for example in the area of physical activity, did not hamper agreement of resolutions at the Summit and was instrumental in funding a research centre to collect better data and evidence for what works. Health officials who recognised the lack of an evidence base for interventions sought to promote those that seemed most logical and appropriate, along with a concern to ensure subsequent careful evaluation. The Summit demonstrated that policy action will move forward in the absence of strong research evidence if government sees the need to respond to public concerns.

However, lack of compelling evidence for interventions is likely to have been a factor in the failure of government to commit significant new funds and to agree to controversial recommendations around food advertising given strong industry opposition. The only contentious resolution taken up by government was for mandatory guidelines for school canteens: this appealed to many community groups and parents who attended the Summit and government is likely to have perceived strong public support for this intervention. Other commentators have questioned the soft policy options adopted in response to the obesity epidemic in Australia following the NSW Summit and raised questions about the way public health issues, such as obesity, are framed in public discourse [31].

The food and advertising industries who were represented at the Summit used the lack of well supported 'scientific' evidence to oppose controls on advertising. In contrast, the debates and resolutions around physical activity using anecdotal evidence, expert opinion and common-sense solutions garnered widespread support as there was no industry that stood to suffer financially from the action proposed. Where strong interests and powerful groups oppose policy direction, the evidence base required for government action, if it is to proceed, needs to be substantial. It is also possible that the more prominent role of the federal government in food advertising regulation and control worked against the agreement of concrete resolutions around food advertising to children.

Economically important industries have been seen by others as critical in the preparedness of governments to support controversial public health initiatives [32] and calls for more research have been presented as tactics to delay policy change [33]. However, creative and clear communication of the evidence has been instrumental in other areas, notably the successful efforts to ban tobacco advertising in Australia and in many other countries around the world despite powerful industry opposition [32, 33]. There is also more scope for interaction and collaboration with the food industry than with the tobacco industry as food as a product is not inherently harmful [33]. The food industry can have an important role in supporting a range of policy initiatives that promote healthy eating as was evident in the NSW Childhood Obesity Summit, but are likely to remain adversarial where industry profits are, or appear to be, at stake.

## Conclusion

The NSW Childhood Obesity Summit played a role in promoting an agenda for action to address childhood obesity. It raised awareness in the public and political arena and provided a public forum for debating research evidence. The Summit demonstrated that while it is not necessary to have all the evidence in place to agree actions, that more radical policy change is much more difficult to achieve in the absence of established and detailed evidence, given the interests of important stakeholders, notably the private sector. The process and the outcomes of the Summit suggest that in the absence of strong Type 1 data, and where Type 2 evidence is contested, that policy-makers may opt for the path of least resistance: a call for more and better research and support for the systematic evaluation of interventions. While beneficial to researchers, direct and short term health gain may be limited.

The lack of an agreed evidence-base provides politicians with a freer hand in choosing actions which have wide appeal and are less controversial, rather than those which may produce greatest health benefit. The Summit's success in generating a set of resolutions should not be discounted even if large resource allocations were not forthcoming. Tobacco control initiatives have taken decades of concerted effort to realise [33] and obesity control efforts are likely to face the same challenges around evidence and action. The prospects of controlling obesity in the future will be amplified if researchers and public health advocates enhance their understanding of the policy process, the interests and tactics of the different stakeholders involved, and the role different types of evidence can play in influencing public debate and the decisions of policy-makers in time-limited yet high profile events such as Summits. Further research is needed to increase our understanding of the role of Summits in the broader politics and processes of policy-making.

## Competing interests

Elizabeth Develin was involved in the original work described leading up to and including the NSW Child Obesity Summit and is employed by NSW Health. All the other authors are part of the School of Public health and Community Medicine, UNSW, which is in receipt of modest funds to collaboratively develop this reflection and paper by NSW Health, the body which organised the Summit described.

## Authors' contributions

This paper was conceived jointly by all authors; all contributed to analysis and writing. SN prepared the first draft with significant contributions by ED and comments from AZ and NG. All authors contributed to writing successive drafts, feeding in literature, analysis and insights.
